# Pre-treatment before coronary artery bypass surgery improves post-operative outcomes in moderate chronic obstructive pulmonary disease patients

**DOI:** 10.5830/CVJA-2013-034

**Published:** 2013-06

**Authors:** Bilgehan Savas Oz, Erkan Kaya, Gokhan Arslan, Kubilay Karabacak, Faruk Cingoz, Mehmet Arslan

**Affiliations:** Gulhane Military Medical Academy, Cardiovascular Surgery Department, Etlik, Ankara, Turkey; Gulhane Military Medical Academy, Cardiovascular Surgery Department, Etlik, Ankara, Turkey; Gulhane Military Medical Academy, Cardiovascular Surgery Department, Etlik, Ankara, Turkey; Gulhane Military Medical Academy, Cardiovascular Surgery Department, Etlik, Ankara, Turkey; Gulhane Military Medical Academy, Cardiovascular Surgery Department, Etlik, Ankara, Turkey; Gulhane Military Medical Academy, Cardiovascular Surgery Department, Etlik, Ankara, Turkey

**Keywords:** cardiac surgery, complication, EuroSCORE, morbidity, risk factors

## Abstract

**Introduction:**

Chronic obstructive pulmonary disease (COPD) has traditionally been recognised as a predictor of poorer early outcomes in patients undergoing coronary artery bypass grafting (CABG). The aim of this study was to analyse the impact of different COPD stages, as defined by the Global Initiative for Chronic Obstructive Lung Disease (GOLD) spirometric criteria, on the early surgical outcomes in patients undergoing primary isolated non-emergency CABG

**Methods:**

Between January 2008 and April 2012, 1 737 consecutive patients underwent isolated CABG in the Department of Cardiovascular Surgery of Gulhane Military Academy of Medicine; 127 patients with the diagnosis of moderate-risk COPD were operated on. Only 104 patients with available pulmonary function tests and no missing data were included in the study. Two different treatment protocols had been used before and after 2010. Before 2010, no treatment was applied to patients with moderate COPD before the CABG procedure. After 2010, a pre-treatment protocol was initiated. Patients who had undergone surgery between 2008 and 2010 were placed in group 1 (no pre-treatment, *n* = 51) and patients who had undergone surgery between 2010 and 2012 comprised group 2 (pre-treatment group, *n* = 53). These two groups were compared according to the postoperative morbidity and mortality rates retrospectively, from medical reports.

**Results:**

The mean ages of the patients in both groups were 62.1 ± 7.6 and 64.5 ± 6.4 years, respectively. Thirty-nine of the patients in group 1 and 38 in group 2 were male. There were similar numbers of risk factors such as diabetes, hypertension, renal disease (two patients in each group), previous stroke and myocardial infarction in both groups. The mean ejection fractions of the patients were 53.3 ± 11.5% and 50.2 ± 10.8%, respectively. Mean EuroSCOREs of the patients were 5.5 ± 2.3 and 5.9 ± 2.5, respectively in the groups. The average numbers of the grafts were 3.1 ± 1.0 and 2.9 ± 0.9. Mean extubation times were 8.52 ± 1.3 hours in group 1 and 6.34 ± 1.0 hours in group 2. The numbers of patients who needed pharmacological inotropic support were 12 in group 1 and five in group 2. Duration of hospital stay of the patients was shorter in group 2. While there were 14 patients with post-operative atrial fibrillation (PAF) in group 1, the number of patients with PAF in group 2 was five. Whereas there were seven patients who had pleural effusions requiring drainage in group 1, there were only two in group 2. There were three mortalities in group 1, and one in group 2. There were no sternal infections and sternal dehiscences in either group.

**Conclusion:**

Pre-treatment in moderate-risk COPD patients improved post-operative outcomes while decreasing adverse events and complications. Therefore for patients undergoing elective CABG, we recommend the use of medical treatment.

## Abstract

Chronic obstructive pulmonary disease (COPD) is one of the leading causes of chronic morbidity and death in the world and it has traditionally been recognised as a predictor of poorer early outcomes in patients undergoing coronary artery bypass grafting (CABG).^1^,^2^ The EuroSCORE system also includes chronic lung disease as an independent predictor of operative mortality, although with a generic definition not necessarily reflecting disease severity.^3^ By contrast, some recent studies deny the association between COPD and increased early morbidity and mortality risk after CABG. Given the heterogeneity of clinical and/or spirometric variables used to define COPD by these different authors,^2^ and the continuous emphasis recent guidelines place on the importance of spirometry as the gold standard for the diagnosis and staging of severity in COPD patients (GOLD guidelines update available at http://www.goldcopd.org),^4^ further investigation on this topic was deemed necessary.

Post-operative complications such as respiratory failure, prolonged intubation time, intensive care unit (ICU) and hospital stay, sternal dehiscence and post-operative rhythm disturbances (mainly atrial fibrillation) are common in COPD patients.^1^ Standard median sternotomy and cardiopulmonary bypass (CPB) have negative effects on pulmonary function. Pleurotomy during the harvesting of the left internal thoracic artery (LITA) and pain (due to chest tubes and incisions) may also negatively affect the patient’s lung capacity. Patients with moderate COPD already have limited lung capacity and these patients may be severely affected by the effects of both CPB and the surgical trauma.^5^

The purpose of this study was to analyse the impact of pre-treatment in moderate COPD patients, as defined by the Global Initiative for Chronic Obstructive Lung Disease (GOLD) spirometric criteria,^4^ on early surgical outcomes in patients undergoing primary isolated non-emergency CABG.

## Methods

Between January 2008 and April 2012, 1 737 consecutive patients underwent an isolated CABG operation in the Department of Cardiovascular Surgery of Gulhane Military Academy of Medicine. Patients were identified from a prospectively maintained surgical database, and medical charts were reviewed retrospectively. Of these 1 737 patients, 127 with a diagnosis of moderate-risk COPD were operated on. Of these 127 patients, only 104 with available pulmonary function tests and no missing data were included in the study. Exclusion criteria were significant valve disease, emergency operation, and approaches other than median sternotomy, and surgical procedures other than CABG.

According to the practice of the Department of Pulmonary Diseases, two different treatment protocols were used before and after 2010. Before 2010, no treatment was applied to patients with moderate COPD before the CABG procedure. After 2010, a pre-treatment protocol was initiated (inhaled bronchodilator and steroid treatment for 10 days before the day of surgery). Patients who had undergone surgery between 2008 and 2010 comprised group 1 (no pre-treatment) and patients who had undergone surgery between 2010 and 2012 made up group 2 (pre-treatment group). These two groups were compared according to the postoperative morbidity and mortality rates retrospectively, from medical reports.

According to the GOLD COPD 2011 guideline, COPD was defined as a common, preventable and treatable disease, characterised by persistent airflow limitations, which are usually progressive and associated with an enhanced chronic inflammatory response in the airways and the lung to noxious particles and gases. Clinical diagnosis of COPD should be considered in a patient who has dyspnoea, chronic cough, or sputum production and/or a history of exposure to risk factors for the disease.

Spirometry is required to make the diagnosis in this clinical context; the presence of a post-bronchodilator and FEV_1_/FVC < 0.70 (forced expiratory volume/forced vital capacity) confirms persistent airflow limitations and therefore COPD. According to the GOLD COPD 2012 guideline, the severity of airflow limitations in COPD is classified into four levels. In this classification, moderate COPD patients are defined as 50% ≤ FEV_1_ < 80%, predicted in patients with FEV_1_/FVC < 0.70.

A pulmonary function test (spirometry) was performed according to the previously described guidelines. FEV_1_, FVC and FEV_1_/FVC were expressed according to the reference values published by the European Respiratory Society in 1993. All surgical records were reviewed to determine the surgical procedure performed, cardioplegic technique, cross-clamp time, cardiopulmonary bypass times, number of grafts, left internal thoracic artery (LITA) usage, and number of blood products used.

An isolated CABG procedure was performed in all patients. Standard anaesthesia and surgical technique, extracorporeal circulation and myocardial protection methods were used. A median sternotomy approach was done in all patients. CPB was installed through the ascending aorta and right atrial cannulation and it was performed with roller pumps and membrane oxygenation. Myocardial protection was intermittent cold blood cardioplegia. All patients received antegrade cardioplegia and ‘hot-shot’ (reperfusion with warm cardioplegia). The lowest core temperature was between 28 and 32°C, depending on the surgeon’s preference.

The patients were transferred to the ICU just after the operation and they received ventilator assistance and monitoring. Extubation was undertaken when the patient’s criteria were stable, and time to extubation was also recorded.

The primary outcome was post-operative mortality in hospital and at 30 days. Secondary outcomes included the length of hospital stay, length of stay in ICU, time to extubation, re-intubations, pulmonary infections, pneumothorax, pleural effusions, atrial fibrillation, other arrhythmias, mediastinitis and sternal dehiscence, need for inotropic support, and low-cardiac output syndrome (LCOS).

Pulmonary infections included pneumonia and bronchitis. Pneumonia was defined by radiological evidence of new infiltration, consolidation or cavity, and antibiotic usage in the presence of one of the three following criteria: purulent sputum, positive blood culture or positive bronchial secretion culture. Bronchitis was defined by the presence of purulent sputum production and antibiotic use. Pleural effusion was included in the analysis only if it required drainage during hospitalisation. Arrhythmias other than atrial fibrillation included supraventricular arrhythmias, atrio-ventricular block requiring pacemaker, ventricular tachycardia, ventricular fibrillation and asystole. LCOS was considered when postoperative inotropic support was used for more than 24 hours.

## Statistical analysis

Statistical analysis was performed with SPSS 15.0 for Windows. Continuous data were presented as mean ± SD. Nominal data were presented as frequencies and percentages. Differences were analysed with Levene’s test, Fischer’s exact test, the Mann-Whitney *U*-test and chi-square test.

## Results

The mean ages of the patients in both groups were 62.1 ± 7.6 and 64.5 ± 6.4 years, respectively. Thirty-nine of the patients in group 1 and 38 in group 2 were male. Mean FEV_1_ values of the patients in both groups were 46.1 ± 2.3% and 48.2 ± 2.1%, respectively. Mean ejection fractions of the patients were 53.3 ± 11.5% and 50.2 ± 10.8%, respectively. Mean EuroSCOREs of the patients were 5.5 ± 2.3 and 5.9 ± 2.5, respectively in both groups. There were similar risk factors in both groups, such as diabetes, hypertension, renal disease (two patients in each group), previous stroke and myocardial infarction. Demographic details of the patients are summarised in Table 1.

**Table 1.** Patient Characteristics

*Variable*	*Group 1 (n = 53)*	*Group 2 (n = 51)*	p*-value*
Age (years)	62.1 ± 7.6	64.5 ± 6.4	0.856
BMI	27.7 ± 3.1	28.2 ± 2.7	0.943
Gender			
Male	39	38	0.842
Female	14	13	0.911
Hypertension	32		0.932
Diabetes	17	35	0.731
Renal disease	2	20	1
NYHA class	1.9 ± 0.7	2	0.911
Mean FEV_1_ (%)	46.1 ± 2.3	2,0 ± 0.8	0.823
EF (%)	53.3 ± 11.5	48.2 ± 2.1	0.678
Previous MI	23	50.2 ± 10.8	0.956
Previous stroke	3	24	0.745
CRF	2	2	1
EuroSCORE	5.5 ± 2.3	2	0.821

BMI: body mass index, NYHA: New York Heart Association, FEV: forced expiratory volume, EF: ejection fraction, MI: myocardial infarction, CRF: chronic renal failure.

With regard to the primary outcome, there were three mortalities in group 1, and one in group 2. The causes of death included cardiogenic shock (*n* = 2), sepsis and multi-organ failure (*n* = 1), and cerebrovascular accident (*n* = 1).

There was no statistically significant difference between the groups with regard to CPB time, cross-clamp time and average graft numbers. However, when we looked at the mean extubation times, there was a statistically significant difference between the groups. Extubation times were shorter in group 2 (group 1: 8.52 ± 1.3 and group 2: 6.34 ± 1.0 hours; *p* = 0.032).

While there were seven patients who had pleural effusions requiring drainage in group 1, there were only two patients with pleural effusions requiring drainage in group 2 (*p* = 0.044). Whereas there were 14 patients with post-operative atrial fibrillation (PAF) in group 1, the number of patients with PAF was five in group 2 (*p* = 0.031). All PAF patients excluding two were converted medically (amiodarone) to sinus rhythm in group 1. The remaining two were converted to sinus rhythm by D/C cardioversion. In group 2 all five patients with PAF were converted to sinus rhythm medically.

The number of patients who needed pharmacological inotropic support was 12 in group 1 and five in group 2. Pulmonary infections such as pneumonia were more frequent in group 1 compared to group 2. There were no sternal infections or sternal dehiscence in either group. The duration of ICU and hospital stay was shorter in group 2. Post-operative data of the patients are detailed in Table 2.

**Table 2.** Post-Operative Variables Of The Patients

*Variable*	*Group 1 (n = 53)*	*Group 2 (n = 51)*	p*-value*
Cross-clamp time (min)	67.2 ± 16.7	65.4 ± 19.2	0.453
CPB time (min)	140.5 ± 43.6	135.3 ± 50.4	0.654
Average number of grafts	3.1 ± 1.0	2.9 ± 0.9	0.744
Extubation time (h)	8.52 ± 1.3	6.34 ± 1.0	0.032
Re-intubation	1	–	0.5
Sternal dehiscence	–	–	–
Wound infections	1	–	0.5
Pleural effusions	7	2	0.044
Pneumonia	4	1	0.171
Mediastinitis	–	–	–
Atrial fibrillation	14	5	0.031
Other rhythm disturbances	–	–	–
Inotropic support	12	5	0.029
IABP support	3	2	0.742
LCOS	–	–	–
Length of ICU stay (days)	2.4 ± 1.2	1.4 ± 1.1	0.039
Length of hospital stay (days)	12.95 ± 2.4	8.29 ± 1.7	0.028
30-day mortality	3	1	0.302

CPB: cardiopuolmonary bypass, IABP: intra-aortic balloon pump, LCOS: low-cardiac output syndrome, ICU: intensive care unit.

## Discussion

CABG is a safe and effective surgical treatment that is performed successfully in a wide variety of patients.^1^ Nowadays the profile of patients undergoing CABG is changing to a higher-risk profile; elderly patients with co-morbid medical problems. With improved experience, cardiac risk factors such as left main coronary artery disease and angina class have lost their predictive value for mortality in favour of extra-cardiac factors such as peripheral vasculopathy, chronic renal failure or COPD.^6^,^7^

The impact of COPD in patients undergoing open-heart surgery is potentially problematic because of the additional influence of CPB and median sternotomy.^8^ It is well known that CPB interferes with pulmonary function. CPB can also induce adverse effects on alveolar stability by activating the complement system, sequestration of neutrophils in the pulmonary vascular bed, releasing oxygen-derived free radicals and changing the composition of alveolar surfactant.^8^ Atelectasis is one of the most important problems after CPB, especially in the early postoperative period.

Median sternotomy also has a negative influence on pulmonary function. Structural changes in the chest wall after sternotomy are the cause of restrictive pulmonary dysfunction, which can be prolonged for weeks after the operation. Lung injury becomes more prominent after surgery in COPD patients. Therefore COPD has been established as an important risk factor for mortality in patients undergoing CABG.^7^

Depending on the severity of the pulmonary dysfunction, the morbidity and mortality of the procedure can be very high and sometimes almost prohibitive. Therefore a correct diagnosis and defining its severity is mandatory because it could allow better planning strategies.^9^ In high-risk patients, it is imperative to institute vigorous pre-operative measures to improve the respiratory status before the surgical procedure. The degree of severity of these risk factors has an important prognostic relevance and not the risk factor itself. Mild COPD is well tolerated by CABG patients in comparison with moderate or severe COPD. As indicated by Fuster *et al*.,^7^ FEV_1_ must be the reference variable when a patient with COPD is considered for CABG, as is the creatinine level for chronic renal failure patients.^7^

Morbidity due to COPD usually increases with age and is higher in males than females.^10^ In their study, Fuster *et al*.^7^ reported that the mortality rate was 13% in patients over 75 years, while it was 7% in patients under 75 years. In our study, the patient population was on average 65 years, which was younger than Fuster’s patient population, and we had three mortalities in group 1 and one in group 2.

Adverse respiratory system events such as respiratory failure and pneumonia have traditionally been the leading cause of post-operative complications.^11^ COPD patients particularly are at an increased risk for lower respiratory tract infections because of the immune-suppressing effects of CPB, combined with the respiratory flora of these patients.

As demonstrated by Gaynes *et al*.,^12^ development of pneumonia following CABG in COPD patients was associated with a 27% mortality rate. In the study by Fuster *et al*.,^7^ 18% of their moderate-to-severe COPD patients had post-operative pneumonia, with a mortality rate of 56%. In another study, Manganas *et al*.^13^ reported more frequent pneumonia in COPD patients than in the control group (eight in the mild-to-moderate COPD group and two in the severe COPD group). In our study we had four pneumonia cases in group 1 and one in group 2.

Moreover, prolonged ventilation is known to result in increased ICU stay. Although in their study, Manganas *et al*.^13^ found no difference between their study groups for prolonged mechanical ventilation and length of ICU stay, in our study, mean mechanical ventilation times and length of ICU stay of patients in group 1 were significantly longer than in patients in group 2. Similar to our findings, in Fuster’s study,^7^ incidence of prolonged ventilation and re-intubation was higher in moderate-to-severe COPD patients.

In a study by Bingol *et al.*,^1^ the effect of prophylactic oral prednisolone in COPD patients was assessed. As a result they demonstrated that prophylactic treatment with prednisolone decreased both mechanical ventilation time and length of stay in ICU. These results were similar to our findings. From this point of view, it can be extrapolated that pre-treatment before surgery improves post-operative pulmonary function and shortens ICU stay.

Supraventricular tachyarrhythmias are common after CABG in COPD patients. In their study, Manganas reported 30% atrial fibrillation in the mild-to-moderate COPD group and 45% in the severe COPD group.^13^ In Fuster’s study,^7^ the incidence of atrial fibrillation was lower than in the study by Manganas. The rate was 7.6% in the moderate group and 11.4% in the severe COPD group. In our study, there were 14 (26%) patients with atrial fibrillation in group 1 and five (9%) in group 2. For group 1, our results were similar to Manganas’s study. From this, it can be concluded that the rate of atrial fibrillation was significantly lower in the pre-treatment group.

Optimisation of management in the pre-, peri- and postoperative periods may be the key to reducing the negative outcomes in this high-risk group.^14^ It is important to improve the respiratory status of these patients by means of adjustment of their bronchodilator therapy and strict control by a physiotherapist. The correct timing of surgery is also mandatory in order to avoid the respiratory decompensation phases.^7^ In the present study, we found that pre-treatment before surgery in moderate COPD patients improved early post-operative outcomes and decreased complications following CABG.

## Conclusion

Pre-treatment in moderate-risk COPD patients improved postoperative outcomes while decreasing the adverse events and complications. We believe that in order to improve post-operative outcomes, a holistic approach must be applied for these patients. Not only bronchodilator treatment but also appropriate antibiotic treatment, besides physical exercise under strict control and perfect timing are key factors for the best results following CABG.
